# Supplementary material for: Informed consent and trial prioritization for clinical studies during the COVID-19 pandemic. Stakeholder experiences and viewpoints
Source: PLoS One. 2024 Apr 30;19(4):e0302755. doi: 10.1371/journal.pone.0302755 (PMC11060594; doi:10.1371/journal.pone.0302755)
Supplement: S1 Table — (PDF) [file pone.0302755.s001.pdf]

Supplementary Table S1
Interview Topic Guide

Introduction– General Remarks

- The funder for this project is the Federal Ministry of Education and research (short in German: BMBF)
- The Principal Investigator is Daniel Strech, and the research team consists of members trained in ethics, regulatory sciences, and clinical care
- We will be asking you a series of questions; please answer as freely and honestly as you can
- The interview will take about 45 minutes and will be recorded so that we can analyze the text later
- Do you have any questions?
- [Make sure you already received a signed consent form]
- [Start recording]

Introduction – Remarks about the project

- We are conducting a BMBF-funded study dedicated to the ethical-legal dimensions of clinical research on COVID-19 patients.
- In our work to date (including a literature review and a survey of German ethics committees) and in discussion with our external advisory board, two topics have been identified as particularly urgent and of broad relevance: **Informed consent and prioritization of clinical trials.** Our project aims to develop recommendations for action on both topics.
- It is particularly important to us that this is done in close collaboration with stakeholders from the field. We therefore work on two levels: on the one hand, we develop an ethical-legal analysis together with lawyers - in the sense of a theoretical foundation. On the other hand, we conduct interviews with experts who are involved in clinical COVID-19 studies at different levels.

<u>Informed Consent</u>

- I would like to start with the topic of informed consent. Before we get to your experiences, could you first describe how you are involved with informed consent processes in your position?

What do you think are the key challenges in the informed consent processes for COVID-19 clinical trials in the pandemic?

➔ Possibly add:

o Were there any particular changes in contrast to the pre-pandemic routine?

➔ if not already a topic:

What were the organizational challenges?

o To what extent did the isolation requirements make informed condent procedures, or recruitment, more difficult?

o How did you and your team handle this?

o If applicable, concise examples? Positive and negative

o What was the impact of staff shortages and redistribution of teams?

o Were study nurses removed? Did you had non-specialists on the team?

➔ if not already a topic or to go into more depth:

Dealing with patients who are no longer (adequately) able to give consent.

o How did you and your team deal with study inclusions - or with the clarifications - in the case of patients who were no longer (adequately) able to give consent? (i.e. severe respiratory distress, delirium, already intubated)

o Possible topics: Legally designated representative? Consultation procedure? Subsequent consents? Rapid clarification shortly before intubation?

o Were there any conflicts in the course of this that were discussed by the team?

o Can you approximate how often unresponsive/incapacitated COVID-19 patients were potential study candidates?

Prospective Consent/Information

o Was there an attempt to inform COVID-19 patients at an early stage - before respiratory decompensation - about the possibility of participating in the study?

o What would be the advantages and disadvantages of such prior information?

Ethics committees

o Have there been challenges in recent months with ethics committee approval of study applications due to "consent issues"?

o Can you describe these in more detail?

Modification of standards

- A pandemic like COVID-19 is in many ways a special situation with a national health threat, so there has been a lot of pressure in a short period of time to generate evidence-based knowledge in the treatment of COVID-19. Our project would therefore also like to address the question of where the normally applicable standards for informed consent (which are already difficult in the critical care setting) could or perhaps even should be softened somewhat in order to be able to conduct important studies even more effectively and efficiently.

  o At what points would this be conceivable and possibly helpful?
  o What would you like to see (for tradeoffs)?
  o What would there be accompanying protective measures? (for patients as well as for the clinical team)

<u>Prioritization</u>

- The second key issue addressed in our study concerns the experience and practice with the high number of COVID-19 clinical trials that were simultaneously brought to or developed at the university hospitals/ clnical study centers.
- The initial analyses of the project indicate that, regardless of the "approval status" by ethics committees, there must have been trade-offs between COVID-19 trials, both in terms of initiations and in terms of subsequent recruitment processes.
  Have you come into contact with this issue at your center?

Organization

o Were these decisions organized in any way at your university? Was there a task force, study board, steering committee or something similar?
o Who was involved in the decision-making process?
o What aspects guided the decisions?

What do you think are the key challenges in prioritizing COVID-19 studies?

o What types of resources lead to prioritization? (e.g. limited patient numbers, familiar collaborations/networks, looking at what the center can use to "raise" its own profile?)

o Were there any particular conflicts that affected this issue?

Final questions:

- Anything else you think we need to consider moving forward? Anything we missed?
- Suggestions for colleagues who would be interesting as further interview partners for our project?
