# Supplementary material for: Informed consent and trial prioritization for clinical studies during the COVID-19 pandemic. Stakeholder experiences and viewpoints
Source: PLoS One. 2024 Apr 30;19(4):e0302755. doi: 10.1371/journal.pone.0302755 (PMC11060594; doi:10.1371/journal.pone.0302755)
Supplement: S2 Table — (PDF) [file pone.0302755.s002.pdf]

Supplementary Table S2

Part "Informed Consent":
Qualitative spectrum of topics with relevance for the informed consent for clinical studies during the Covid-19 pandemic

Main categories and first-level sub-categories	Second-level sub-categories	#	Quotes from anonymized interview transcripts
Consent challenges			
Time pressure	Rapid deterioration of the patient's condition	C1	If you are the only person who can enroll a patient in a trial today, you have to do it today, because tomorrow the patient may not be able to give consent, or it may be too late. (Interview 14)<br><br>The most important points are how to deal with patients who are unable of giving consent, who may be able to give consent at the time of enrolment or screening, but who become unable of giving consent during the course of the disease because their condition deteriorates, for example because they need ventilation or intensive care and so on.. (Interview 8)<br><br>So if someone is severely ventilator-dependent and barely alive, that's obviously a situation where you don't really want to tell anyone about the trial enrolment, but you need the decision, don't you? (Interview 5)
	Reduction of contact time	C2	[...]When educating patients in the field, you want to minimise the amount of material that goes in and out. And, of course, you also want to reduce the contact time inside and the frequency of going in and out, or keep it as low as possible. (Interview 6)
Isolation, physical distancing, and hygiene conditions	Difficulty in building the doctor-patient relationship	C3	The particular challenge with a highly infectious disease, and this does not have to be in the context of a pandemic, is of course that it is less easy to give the patient the impression that you are now going to sit down with them in peace and have a personal conversation. If I come to a patient in full disguise and the patient can only see my eyes through a plexiglass window, it is of course difficult to create a trusting basis for a partnership discussion. (Interview 1)
	Need to reduce the number of patient contact	C4	And of course you also want to [...] reduce the frequency of entry and exit, or keep it as low as possible. (Interview 6)
	Restricted accessibility to legal guardians due to quarantine regulations	C5	And what has been taken into account, or what is of course also a problem, is now also in the context of the pandemic, that of course even if there are legal guardians, they are of course quarantined by the pandemic or even in quarantine. (Interview 12)
	Contact restrictions for relatives	C6	And then, of course, the problem of not having relatives within reach, which was easier before the pandemic because of the hygienic conditions - those were really the challenges. (Interview 17)

Overburdening the patient	Distress due to parallel/competitive study recruitments	C7	For example, doctor A goes to the patient and asks: "Would you like to take part in the XY drug trial? The patient asks for time to think about it. During the reflection period, Doctor B comes and clarifies for the next study. And the patient thinks it's the same trial and says yes. These things can happen. (Interview 6)
	Difficulties in understanding due to complexity	C8	[...] and it's gotten so complex that I'm always worried about whether the person who's not an expert can really do anything with the information, or if it's just too overwhelming for them to sign. (Interview 10)<br><br>The documents are often 10 or 12 pages long. That's too long, and they're presented in such a complex way that the patient can't understand them. (Interview 4)<br><br>Many were also sceptical (about participating in a study for a foreign disease such as COVID-19) [...] they were often in a very bad condition and then overwhelmed by all the information you have to give them quickly. (Interview 7)
	Restrictions of the patients due to severe symptomatology	C9	The biggest challenge now in terms of trials was simply getting consent, because the patients were already breathing quite hard and had difficulty just thinking about it calmly. (Interview 7)<br><br>Even if you now say: "I only accept patients who first come to a normal ward and are still healthy enough, in quotes, to be easily educated", there will still be some who are short of breath, anxious, confused or whatever. (Interview 1)
	Increased risk of therapeutic misunderstanding	C10	[...] this situation of "I'm coming into intensive care and I'm afraid of what's coming and what's going to happen" [is] threatening, and then someone else comes along and says "I can help you, but you have to sign for me once". That is, of course, extraordinarily difficult. (Interview 1)
Impact of consent challenges on clinical research			
Heterogenous ethics review		C11	For example, in our drug trial, we were told that only patients who were able to give consent could be included. This means that as soon as they [those who have already given their initial consent] are no longer able to consent, or as soon as they are intubated, they are drop outs. (Interview 14)<br><br>The question is: what do I do with the patient who goes to intensive care? The hospital ethics committee has said: "In the observational study, you can just leave him there, even if he's sedated and ventilated in the meantime, and then he can't withdraw his consent". The government agency says, or a person in the government agency says, "The moment the patient goes into intensive care, the patient has to be excluded from the trial". Although, of course, you could just as easily say, "Well,

			he's consented, he wants to stay in the trial, even though he can't say anything about it now. But because he can no longer withdraw, you have to exclude him. (Interview 1)
Delay		C12	We still have an insane number of clinical trials, and now we have that situation again: At the moment, none of the centres are recruiting very well because there are hardly any patients left. We must not forget that if the protocols are complicated, the inclusion and exclusion criteria will not work. (Interview 9)
Recruitment failure		C13	We still have an insane number of clinical trials, and now we have that situation again: At the moment, none of the centres are recruiting very well because there are hardly any patients left. We must not forget that if the protocols are complicated, the inclusion and exclusion criteria will not work. (Interview 9)
Bias	Excluding critically ill patients introduces important bias	C14	[...] if someone is unable to give consent at the beginning of the trial: For these patients, I think you have to do everything you can to include them in the trials, because otherwise you get an enormous bias if you leave out the seriously ill. Then, of course, the significance is much, much lower. (Interview 16)<br><br>That's where a completely different argument comes in: The moment you eliminate the seriously ill, i.e. discard the material, you introduce a bias, and then it is no longer representative at all - you need the seriously ill. (Interview 16)
Potential strategies for consent challenges			
Opt-in consent as ethical standard for interventional studies	Relevance for interventional studies	C15	I think everyone would fight tooth and nail against saying that informed consent should be abolished. (Interview 10).<br><br>Then I think in drug trials there always has to be some kind of consensus: I think that's important. (Interview 14).
	Conflicts of interest	C16	Never [change consent in times of crisis]! Doctors always have conflicts of interest. (Interview 3)
Consent models for secondary use of patient data	Broad consent model	C17	We put a lot of emphasis on the fact that in these two cohort studies, staff and patients, these are the two cohorts, both broad consent... so there were two separate things created. A broad consent biobank and then a registry. This allowed us to collect virtually all the routine data that was generated. We made sure that patients signed the correct consent forms, which then allowed us to re-contact them and so on. (Interview 16)
	Opt-out model	C18	Exactly with such a, let's say, medical or such an inclusion procedure and then, so to speak, with such an opt-out solution, I would not find it so strange for register studies. (Interview 14)
	No consent model	C19	Intervention study versus data summary. And that's where I first come to the question of the availability and use of patient data for later use and also for biomaterials. And I have to say, for this part, my impression at Covid-19 was that the interest for the general public is so great that it would

			be helpful to define at a high level what is generally possible for the general public and does not require consent. (Interview 11)
Requirements for patients unable to give consent	Need of consistent IRB requirements	C20	The question is: what do I do with the patient who goes to intensive care? The hospital ethics committee has said, "In the observational study, you can just leave him there, even if he's sedated and ventilated in the meantime, and then he can't withdraw his consent". The government agency says, or a person in the government agency says, "The moment the patient goes into intensive care, the patient has to be excluded from the trial". Although, of course, you could just as easily say, "Well, he's consented, he wants to stay in the trial, even though he can't say anything about it now. But because he can no longer withdraw, you have to exclude him. (Interview 1)
	Deferred and legal proxy consent	C21	Well, I would not necessarily require deferred consent, but a combination of deferred consent and a consultant might make it better, yes; that someone from a different field actually has to look at it. I think in observational studies it's relatively uncritical and in interventional studies I think it's also appropriate. (Interview 1)<br><br>[...] a husband or wife and they have an acute event and they have to go to intensive care and they have to be ventilated and they could be in a trial. Then the partner usually has to sign. But he is completely overwhelmed with the current situation, so I would delegate more to the doctors for those intensive care studies where I simply have the know-how - it is such complex medicine. (Interview 4)
	Alternative proxy decision makers	C22	[...] a husband or wife and they have an acute event and they have to go to intensive care and they have to be ventilated and they could be in a trial. Then the partner usually has to sign. But he is completely overwhelmed with the current situation, so I would delegate more to the doctors for those intensive care studies where I simply have the know-how - it is such complex medicine. (Interview 4)<br><br>And this is something that we have seen again and again with the Covid patients, in the sense that - now there is the issue of group benefit - in this pandemic, in this danger for the entire population, the ethics committee has decided that if someone is no longer capable of giving consent and there is no caregiver present and there is no objection because a relative says 'no', then the ethics committee takes responsibility so that biological samples can be taken for research as soon as a patient arrives at the clinic. (Interview 16)<br><br>(c) Exactly, that [the inclusion of seriously ill, non-consenting patients in clinical trials] compromises the scientific validity - that is a gross mistake in my eyes. And for that you need an ethics committee, you see? You need an ethics committee that allows you to do that. (Interview 16)

Part “Prioritization aspects”:
Qualitative spectrum of topics with relevance for the prioritization of clinical studies during the Covid-19 pandemic

Main categories and first-level sub-categories	Second-level sub-categories	#	Quotes from anonymized interview transcripts
Perceived benefits of prioritization			
Lack of explicit prioritization		P10	It's [requests to participate in or recruit for clinical trials] completely unstructured. You're offered something, you don't have anything, and then you just take it, and sometimes there's even overlap. So it's problematic: We have multiple trials that are looking at the same patient population, and that's a problem. (interview 5)<br><br>We have often said that there is no point in doing this study alone: Either we leave it or we join a centre elsewhere.. (Interview 8)
Ensuring patient protection	Prioritization could improve risk-benefit assessment for clinical trials	P1	[...] And then came the reconvalescent plasma trial. It is still running. And the impression is: [...] Not only are we not making a 'benefit', but we are prolonging the intensive care stay. We are rather creating problems. And finally, piece by piece, the studies came here that showed: "It doesn't help there. It doesn't help there. [..] When do you stop such a study? (Interview 11)
	Prioritization could prevent overburdening of patients	P2	[...] the coordination and prioritisation of research projects at the interfaces of the various wards and disciplines, [...] all colleagues [...] want to do interventional studies, five pharmaceutical companies send emails every day wanting to do something. And it has to be said that all this has to be coordinated somehow and a sensible research portfolio has to be created that does not overburden the patient. You can't have people running up to the same patient ten times a day and saying, "I'll tell you about this trial and that trial. That would be questionable in the end. And recognising that the patient's capacity is finite, but that certain things, such as biological

			samples, blood that can be taken, are also finite for certain things, and embedding them in a meaningful portfolio is also a challenge. (Interview 8)
Improving validity and efficiency	Prioritization could reduce the number of parallel/redundant studies	P3	It's [requests to participate in or recruit for clinical trials] completely unstructured. You're offered something, you don't have anything, and then you just take it, and sometimes there's even overlap. So it's problematic: We have multiple trials that are looking at the same patient population and that's a problem. (Interview 5)<br><br>Basically, everyone was expressing their interests in some way, and in the end there was a lack of clarity: "How do you do it if everyone expresses their interests? In the end, is it going to be the case that certain groups, which may have some kind of - let me put it this way - influence, will then perhaps implement their study interests, or how does that work? And in the case of such a pandemic, it would indeed have been helpful if certain therapies or trials or even diagnostic studies had been seen as a priority. (Interview 5)<br><br>We still have an insane number of clinical trials, and now we have that situation again: At the moment, none of the centres are recruiting very well because there are hardly any patients left. We must not forget that if the protocols are complicated, the inclusion and exclusion criteria will not work. (Interview 9)
	Prioritization could improve quality of studies	P4	The quality of protocols needs to improve. The kind of scientific thinking is often simply absurd. […] So many technical mistakes are made. (Interview 16).
	Prioritization could avoid that trials are conducted with insufficient resources and expertise	P5	[...] clinical trials with good, valid data are another area of science that requires resources and expertise. (Interview 9)<br><br>They are afraid that it will take something away from them, but I think the opposite is true: I don't have to do in my clinic what five other clinics are doing in exactly the same way, which is a waste of time and money: Either we do it together or we don't

			do it at all, but I don't have to repeat the same research. (Interview 16)
	Prioritization could reduce inefficiencies due to competitive recruitment	P6	We are not competing with one university town against another university hospital city, for example where we have two university locations here, or in a university hospital city against Berlin, but we are ultimately competing in a large international network. And of course it will not be the case that all 16 universities here have the same experts in the field of infectious diseases, especially as this is still a very small field. But the few that are there have to pull together [...] the relevant stakeholders and somehow moderate and manage them well so that we at least speak with one voice to the public? (Interview 9)
Increasing relevance	Prioritization could support the relevance of clinical studies	P7	[...] this is indeed a problem and I would have liked the national side to have coordinated this and said that these and the studies have priority because they really take us forward and not the Xth study, which examines something but does not really bring a solution in terms of the pandemic. That would have been helpful. (Interview 2)<br><br>It has diminished over time, but when Covid really took off - in March, April, May last year - we actually had several requests a day. And we actually looked at the projects and first of all, is it a scientifically sound concept? Is it clinically meaningful for the patient? And is there a high probability that it will lead to an outcome that is meaningful for the patient or for pandemic research? [...] I would say in order, scientific and clinical sense, secondly resources in the clinic, and thirdly what it might bring in terms of third party funding, in that order. (Interview 8)
	Prioritization could identify patient-oriented knowledge gaps	P8	So for Covid patients in intensive care, ventilation studies were key. How do I ventilate? Zero funding, 0.0 funding from anybody. That means you'll find zero ventilation trials. There aren't any. But for the person in intensive care, it's the most important thing. When do I start ECMO? How long will I be on oxygen? How much hypoxia can I tolerate? There are no trials in the end-stage area, only case reports. (Interview 4)

	Prioritization should include the allocation of biological samples	P9	That's always a good question, of course: Is it necessary and right? And you have to be careful how much material is given to whom? Then the patient is left out in the cold, so to speak. The material is there, the data is there, and then of course you have to look at this limited material: "What research do I think is so important that it can be used for this? (Interview 16)
Potential strategies for prioritization			
Central coordination and collaboration	National board	P11	Well, the one you hope for the most, of course, when you read about how the data situation was really the best. But of course you have to say that it would have been really helpful, as people have said in the past, if a committee had been set up very quickly and had had time to really get to know each other. Maybe even nationwide and would have said: "These are really the best candidates. These are the ones we're counting on". So ultimately through our own research. And then: How feasible is that? Well, there are still a lot of requests. (Interview 11)
	National cooperation	P12	And I actually believe that at the beginning of the pandemic in Germany we were probably in a particularly bad position in an international comparison because of our very decentralised structures and also, I would say, because we are proud of our decentralised structures. (Interview 15)<br><br>We are not competing with one university hospital city against another university hospital city, for example where we have two university locations here, or in a university hospital city against Berlin, but we are ultimately competing in a large international network. And of course it will not be the case that all 16 universities here have the same experts in the field of infectious diseases, especially as this is still a very small field. But the few that are there have to pull together, and that is perhaps one of the merits of Mr X, who is responsible for the national guideline, that he brings together the relevant stakeholders and can somehow moderate and manage them well, so that we at least speak with one voice to the public? (Interview 9)

	Central registry	P13	I think it would be good if the studies - of course it has to be voluntary again, otherwise it won't work - if the individual scientists could report to a central body what studies they are planning. They are afraid that something will be taken away from them, but I think the opposite is true: I don't have to do in my clinic what five other clinics are doing in exactly the same way, which is a waste of time and money: Either we do it together or we don't do it at all, but I don't have to repeat the same research. (Interview 16)
	Consultation by Ad-hoc expert groups	P14	Perhaps temporary competence clusters would be needed, depending on the time and the topic... By competence clusters, I don't mean new institutes, but ad hoc committees made up of different study stakeholders who now come from different institutes and prepare studies in a bundled way. (Interview 18)
	Efficient study planning	P15	But of course, if you want something and you are looking to the future, this process has to be much faster at the European level. In some cases we have had international studies, and that is very, very time-consuming. It really has to be coordinated. In the US, there have been important registration trials in which we in Europe were not even able to participate because we were too slow. There were trials in the UK that we were not able to take part in because the conditions were different. And what was also very unfortunate was that major WHO trials actually passed Germany by again, also because of this very latent and sometimes poorly coordinated process. (Interview 13)
	Effective recruitment of special patient groups	P16	But I think if you really want to generate 'unique' research results from Germany ... We have enough patients. We have 2.5 million patients, right? If you wanted to recruit 'unique' patients, you would have to do it from the top down. (Interview 4)
	Management of conflict of interest	P17	(a) Sure, such a committee would help, but how can such a committee be neutral? (Interview 11)<br><br>Because, of course, as soon as someone is involved in the studies, they are "biased". And if they are not involved, they may not be biased. It's not that simple, I think. But of course you could imagine something like a board of directors. So you could

			say that the management levels of such a committee plus two additional members or so, some real scientists who have distinguished themselves in the field [...]. So they're sort of deeply involved, but they don't have any skin in the game at the end of the day. (Interview 11)
Adapting study design	Multicentre study design	P18	Maybe if we were more multicentric, you would get results much faster. (Interview 14)<br><br>There are three studies running in parallel [...]. And of course it would be helpful to combine them, because the number of cases and the range of people included is getting larger. The background of the participants also depends on whether I am in a practice at the university hospital or in a peripheral hospital, but we have not been able to bring the participants together. (Interview 16)<br><br>We have often said that there is no point in doing this study alone: Either we leave it or we join a centre elsewhere. (Interview 8)
	Adaptive study designs	P19	So if they do, then all they need is a fixed, established treatment group, such as the ACTT [Adaptive COVID-19 Treatment Trial], where a common design is developed under fixed coordination, but there also needs to be a team behind it, because they can then take over the protocol [...] and other tasks in the pandemic - we see all this already with pharmaceutical sponsors. (Interview 9)<br><br>So I miss studies with adaptive designs that can be adapted to the appropriate products and situations. This is sometimes a problem because it means that you also have to look at the extent to which adaptive study designs are actually used. (Interview 18)
Alignment with funding	Funding for priority studies	P20	There is no funding because there is no pharma. There is no funding because there is no virology, no basis in it, but it is a purely clinical issue. I would like to see a central committee. That's where the questions would come from. Then you say

			these are the 'most important' questions for us that need to be answered, you delegate it to the 'disease' PI - if I can call it that - who has to set up a study protocol, and then funding has to come in before everybody does it in their own little box. (Interview 4)
	Calls for priority topics	P21	Well, the one you hope for the most, of course, when you read about how the data situation was really the best. But of course you have to say that it would have been really helpful, as people have said in the past, if a committee had been set up very quickly and had had time to really get to know each other. Maybe even nationwide and would have said: "These are really the best candidates. These are the ones we're counting on". So ultimately through our own research. And then: How feasible is that? (Interview 11)
	Efficient funding procedures	P22	Quite apart from ethics, it is obvious that the funding system in Germany is in no way prepared for a pandemic. In March, 150 million euros were thrown into the pot and people said: "Well, now we have to understand this disease very quickly". And I think the first euro was spent in December. And it wasn't because people didn't want to take the money, but because the mills have to grind in a very, very complicated way because of the legal requirements. (Interview 1)<br><br>The financial support is also so incredibly complicated and takes an incredible amount of time. There is so much bureaucracy behind it. And that is something that is a really big problem for a pandemic, where speed really counts. We got a lot of funding this year, including for clinical trials, but it is incredibly time-consuming to organise that. (Interview 10)
	Fair funding procedures	P23	I would have liked it to be like money. If you want public funding now, you can't do it without *clinic X*. They've got the big money. They got the big money and then you have to make sure that you get a little bit of *clinic X*, right? And [I] find it disastrous that the money went to the virologists and the virologists distributed it mainly to other virologists. So the clinicians, they're completely ... if we didn't have access to study credits through the

			pharmaceutical industry, we wouldn't have done any research at Covid as clinicians, but we would have degenerated into biomaterial suppliers for virologists. And then, of course, you're really interested when you know that you're going to deliver and slave away all day and never be considered anyway. And I thought it was a shame that all the 'public grants' are controlled by *clinic X*, right? […] No, that is because of the pandemic. *Mr X* gets it all and distributes it. And for innovative clinical ideas, you don't get money from public funding, you have to go to the pharmaceutical industry, right? (Interview 4)
	Sunstainable funding	P24	I think a lot of the disruption has come from the fact that, first of all, everyone wanted to contribute to this global issue, but also that a lot of our scientific work today is funded by short-term third-party funding, right? So I now have 31 people in my research group and not one of them is tenured, including myself. So all 32 of us work on temporary contracts and all of us work on competitively acquired third-party funding. (Interview 15)
Pandemic policy on use/ownership of patient data	Agreement for secondary data use	P25	Okay, so if we want to participate here scientifically and above all we just realised that we have something coming up and we actually want to learn while we are 'doing'. So the goal is that we don't say, as we usually do, we're going to collect some data now and evaluate it in a year and have our study protocol, our grant programme or something, but actually more or less every week we want to actually prefer to evaluate what we've learned from the last week because it's just so new, right, and it's coming so fast. (Interview 15)<br><br>Intervention study versus data summary. And that's where I first come to the question of the availability and use of patient data for later use and also for biomaterials. And I have to say, for this part, my impression at Covid-19 was that the interest for the general public is so high that it would be helpful to define at a high level what is generally possible for the general public and does not require consent. (Interview 11)
	Regulation on data use and access	P26	Who owns the data? Am I giving away my data? Am I still considered enough there? So in an instant everybody gets the

			feeling: "I'm giving something away. Am I giving away my rights?" And that somehow seems to be more possible in this NHS [National Health Service], that the individual NHS doctor in the system - even if it's not China now - somehow functions better and immediately feels that something is being taken away from him. (Interview 11)
Professionaliz+ation of clinical research	Competence in clinical research	P27	This is primarily a political issue: How do we deal with the clinical trial landscape in Germany, and my main point of criticism is that there is not only a lack of an appropriate group - that is only a symptom - but it is simply assumed, especially by the very basic-oriented key opinion leaders, that anyone can do clinical trials, and that is just fundamentally wrong in my view. (Interview 9)<br><br>Maybe that's part of the problem, that people are now doing clinical research who haven't really done clinical research in the past, and then the collaborations don't work. (Interview 16)<br><br>And I realise that not every hospital can have a postgraduate training programme, but there would have to be an institutional framework where junior colleagues could go through a curriculum element and then go on to do things effectively on their own. (Interview 18)<br><br>So if I could wish for something, and I would like to turn the situation around, it would be that university policy, but also social policy, recognises that clinical studies need their own scientific basis to meet high quality standards, that the centres need to be equipped accordingly, especially in the field of infectious diseases or, if you look at it more abstractly, in all German centres for health research. (Interview 9)<br><br>It's interesting to see who's doing clinical trials now and who's stopping some of them, so we used to start trials here that never went to recruitment, which is just a cost factor for everybody. (Interview 9)

	Fair play among scientists	P28	So if there is a collaborative body and if you are trying as an institution to make research as efficient as possible in terms of outcomes, but at the same time to keep it as ethical as possible in terms of the participants in the study and also to keep it as resilient as possible, then at the end of the day you also have to make sure that there is fair play among the scientists. (Interview 8)
