# Supplementary material for: Informed consent and trial prioritization for clinical studies during the COVID-19 pandemic. Stakeholder experiences and viewpoints
Source: PLoS One. 2024 Apr 30;19(4):e0302755. doi: 10.1371/journal.pone.0302755 (PMC11060594; doi:10.1371/journal.pone.0302755)
Supplement: Attachment — Submitted filename: PLOS one informed consent and trial prioritisation.docx [file pone.0302755.s003.docx]

This paper addresses an important, interesting, and timely topic. The chosen methods of qualitative research seem appropriate to dig deeper into some preliminary topics identified in a prior literature review conducted by the authors. However, the methods and results section would benefit from further details, while it is also advised that the authors engage more substantively with existing literature in the discussion section. Detailed comments for each section of the paper are provided below. I hope will help to strengthen the impact of this timely paper.

**Introduction:**

“Valid informed consent exists only if the patients concerned were a) competent to make decisions in the situation in question, b) received the relevant information, c) understood it, and d) were free from coercion to decide for or against consent.” Please add a relevant reference to research ethics guidelines (eg. CIOMS, Helsinki) or seminal literature.

“Prioritization of clinical studies that is the ranking of more or less important trials might become relevant, for example, due to a shortage of eligible patients.” The strain/shortage of human resources in the health care sector could be and additional important factor to name here.

“Recent studies showed that many clinical trials, for example in Germany, failed due to insufficient recruitment [5]. Prioritization might also be important if too many studies are conducted in an inefficient or even harmful way. For example, studies showed that many trials investigated the same therapeutic approach at the same time [6].” It seems reference 5 is a literature review of some sort, so say this in text. What kind of study is reference 6 referring to? It would be good to briefly add some context to both these references in text e.g., where these study/these studies were conducted and what they investigated.

“The results of the interview study shall inform the development of practiceoriented guidance for informed consent and trial prioritization as a component of pandemic preparedness.” Can you strengthen this point/argument by specifying which part of pandemic preparedness are you talking about.

**Methods:**

“Based on typical sample sizes in qualitative research and own experiences we estimated that 15-20 interviews are needed to reach thematic saturation on the level of major and first-level subgroup categories.” It seems to me that this sentence reflects more of a quantitative paradigm than a qualitative one. In qualitative research there is no such thing as a ‘typical sample size’ and it is neither necessary nor advisable to state estimated sample sizes, especially when seeking to obtain thematic saturation. Data collection and analysis should be conducted iteratively, continued until saturation is reached. If the authors (for research or time constraints) chose to pre-empt their sample size, estimating it to be 15-20 interviews (as stated here), it should not be claimed the study also adhered to principles of thematic saturation, as this is a contradictory claim. If the former was the case, please remove references to thematic saturation and describe in the limitations section that only this was a rapid qualitative study, in which only 21 interviews were conducted because of time and resource constraints. Particularly given the wide range of stakeholder groups included in this study (clinical investigators, clinicians from clinical trial centers, clinic directors, patient representatives, members of research ethics committees, of regulatory bodies, of public sponsors, of guardianship courts and authorities”) it seems very unlikely that saturation could have been predicted to be reached at 15-20 interviews.

“normal ward” is not a widely used or accepted term. Please specify which kind of ward you are referring to when you say ‘normal’

“We further identified potential interview participants via snowballing.” Please give more detail on who this snowball sample originated from? Was this from those participants already recruited purposively via the QUEST Center, AKEK and PRECOPE?

“We conducted the interviews via videoconferencing…” please specify which videoconferencing platform was used. Please also state somewhere here that interviews were recorded (say if audio or video recorded), as this is currently not explicitly stated (you only mention that they were transcribed).

“MAXQDA 2020 was used for the analysis of the interview material.” Please clarify that the software was used to process and manage the analysis, as the analysis itself cannot be done by the software.

“We used qualitative content analysis according to Mayring (2010) [9].” Please add a sentence or two which summarizes the key components of QCA by Mayring for readers not familiar with this approach.

The data analysis section is rather brief and would also benefit from further details:

- on the research team and reflexivity (including the each of the researcher’s background and positionality and how this may have impacted participant’s responses in terms of social desirability, openness etc.; why the choice was made to have an observer present during interviews and how may this have impacted the interviewer-interviewee dynamics)

It is also notable that three authors, plus one observer each, collected the data but one author did the bulk of analysis. Please provide more details on the following questions:

- Why was this approach chosen rather than analysis within the wider research team?
- What was done with the notes taken by the observers present during interviews? Did these feed into analysis and if so how? If not, what function did the notes serve?
- How did the research team community among themselves to share emerging findings during data collection?
- Was analysis done iteratively or after all data had been collected? If so, how did this impact assessment of saturation, which the authors claim guided them in deciding how many interviews to conduct.

(Later on when reading the author contributions I noted that it says there “SW, AF, DS and LW analyzed and interpreted the data”, please be consistent and clarify how analysis was conducted.)

**Results:**

“thematic (qualitative) saturation” – please remove the word qualitative, the term is thematic saturation

“Altogether, we contacted 51 stakeholders, which gives a response rate of 42%.” Again this sounds very quantatative. In qualitative research we are not really concerned with things like response rate, as it doesn’t really tell us anything at all. Instread, I would suggest simply saying that you contacted and invited 51 people to take part in the study. In case you collected reasons why prospective participants chose not to participate, then you could add these here, as that would be interesting, rather than giving a response rate.

“For the first topic informed consent we identified three main categories” – is there a reason why you refer to categories rather than themes?

“For the first topic informed consent we identified three main categories: consent challenges, impact of consent challenges on clinical research, and potential response strategies for consent challenges. For the second topic prioritization of trials, we identified two main categories: need for prioritization of clinical studies, potential response strategies for prioritization of clinical studies.” – suggestion to remove “for prioritization of clinical studies” as this is quite lengthy for a theme heading and to be consistent with the theme heading under the topic of ‘informed consent’

“first and second level subcategories” – again, why are these not called subthemes?

S2: there are some inconsistencies in the details of reported quotes, with some of them stating “(Interview X)” others “(Interview X, pos. XX)” or “(Interview X, item XX)”. Please ensure there is consistency across the table. If using posX or item X, please explain in a footnote to the table what this is referring to, as it’s not clear to the reader and not a standard way of reporting quotes. Usually, some non-identifiable details of participants (e.g. stakeholder category, gender etc.) are stated after the Interview X, … to give the reader an idea of the positionality of the participant sharing this view.

I like the authors use of direct quotes in text throughout the results section. However, the manuscript would benefit from several full quotes, which exemplify the point being made, from being added in text to the themes being discussed. This would enrich the results section and give readers who do not prioritise consulting table S2 in detail (likely to be most readers!) a flavour of the data and the kinds of views being expressed by participants. I suggest that you add 2-3 key quotes within each of the broad level themes (i.e. 2-3 quotes under Consent challenges, Impact of consent challenges on clinical research and so on…).

The theme of “Isolation and hygiene conditions” seems to also describe conditions of physical distancing more generally, not just isolation. Consider revising title of this theme to reflect that e.g. “Isolation, physical distancing and hygiene conditions”

The theme of “Overburdening the patient” – “Patients often had difficulties in ‘understanding due to complexity’ of informed consent documents. This was further complicated by the pandemic context (C8).” Why and how was this further complicated by the pandemic context? Please explain. (e.g., This is one example of where adding a full quote could help to enrich results and make them more understandable).

Under “Impact of consent challenges on clinical research”

“The challenges around appropriate consent procedures for COVID-19 trials had an impact on conducting clinical research:” – please be careful how you phrase results, as you are not stating facts here but rather reporting on participant’s perceptions of a particular situation. Suggestion to rephrase as something like “Many/some/all interviewees reported that the challenges around appropriate consent procedures for COVID-19 trials had an impact on conducting clinical research”. (similarly with the sentence “‘Sustainable funding’ (P24) becomes important to make the prioritization procedures effective.” later on in the results section – please rephrase so its clear that this represents participants views)

Under “Opt-in consent as ethical standard”: “…some interviewees addressed the protective function of the informed consent for potential ‘conflicts of interest’ of clinician investigators (C16).” Please add more explanation or a quote here, as it’s not clear from this sentence what is meant here.

Theme “Secondary-use of patient data”: this theme contrasts nicely with the “Opt-in consent as ethical standard”, suggesting some difference in views when it comes to intervention studies vs studies of secondary/routine data. Suggestion to adjust the theme heading to make this difference clearer, e.g. by calling it “Opt-out consent for secondary-use of patient data”

Theme “Patients incapable to give consent”: please rethink the title of this theme, as it isn’t very meaningful when considered considering the top-level theme for this category of results, which is about “Potential response strategies for consent challenges”. The title of the theme should be self-explanatory with regards to what the response strategy here is (e.g. opt-in or opt-out), while “Patients incapable to give consent” is not a response strategy.

Theme “Need of prioritization” – it seems to me that this theme is rather describing the “perceived benefits of prioritization”. Consider renaming to make this more self-explanatory. If so, I would also suggest renaming subheadings such as “ensuring patient protection” “improving validity and efficiency” “increasing relevance of trials”. In general, it’s desirable for theme headings to be able to stand alone and make sense on their own, even before reading the detailed results.

Theme: “Lack of explicit prioritization” consider moving this to be right at the top of the section, followed by the other three themes. I think that order would flow more naturally, giving context to the other three themes which focus on the perceived benefits of prioritization.

Theme: “Potential response strategies for prioritization” consider simplifying title to “Potential strategies for prioritization”, as the word response doesn’t add much but makes title unnecessary technical sounding

Theme: “Study design” similar to my above comment about making themes meaningful and self-explanatory, I recommend renaming this into something like “adapting study design”. This theme doesn’t appear very rich. Is there any interesting data the authors could to add to this theme?

**Discussion:**

Section discussing “Informed consent” – there is a great wealth of research ethics literature that has engaged with the question of how to achieve informed consent in research generally and in emergency situations more specifically. I would like to see the discussion on the findings around informed consent engage with some of this literature, in light of the perceptions of the stakeholders shared. For example, what are some of the methods that have been developed in other contexts which could also help to serve informed consent in this setting? A discussion of some of the literature on community engagement and its role in achieving informed consent, as well as on ‘broad consent’ models also seems important here. As it stands, the discussion does not seem sufficiently embedded and engaged with the literature and academic debate on this topic. The authors have done a nice job when discussing the criteria of Meyer et al. [8] in section that followings and should similarly engage with the literature in the discussion on informed consent.

**Limitations:**

Please expand on the first limitation to explain how this limited findings, given you also say it was justified by the literature review.

**General comments:**

In future, please use line numbers on your manuscript submissions, so that reviewers can refer more easily to different sections of the paper, rather than having to copy and paste each sentence. This will make life easier for the reviewer and also for the authors when addressing reviewers comments.

Please follow PLOS ONE style guidelines with regard to second level headings. Currently some second level headings are not clearly visible, for example in the methods section.

S1- topic guide: in places this reads more like a survey, rather than an in-depth interview. For example: “Have you come into contact with this issue at your center?” If yes: check here regarding decisions between studies that were ready in parallel to the initiation and regarding studies that were recruited preferentially” – it is unclear to me what needs to be “checked” here and by whom? Please clarify and correct if this is a typo

- Suggestion for authors to use more open ended questions in future qualitative studies, as these generally yield richer and more unexpected data.

Study lacks the perspective of patients. It would have been interesting to hear from them on their views on trial participation and consent, as surly they are an important ‘stakeholder’ in this topic. The authors mention this in the limitations section but do not give a clear reason for why patients were not included. Please discuss this further in limitations section.

Different stakeholders’ perspectives – I was surprised that the authors did not discuss anywhere in the results and discussion the similarities and differences in views among different stakeholders included in this study. This leaves me wondering why the authors chose to recruite a variation sample if then failing not to compare and contrasting the views of different stakeholders? It is assumed that all stakeholders share the same views? This seems highly unlikely. One characteristic of rich qualitative research is that it can bring out nuanced views and explain them in light of the positionality/perspective of who has shared those views, as well as being able to shed light on deviant findings. Please could the authors respond to this and clarify why different stakeholders’ perspectives are not discussed, or if relevant to revise the results and discussion section accordingly.

**Proofreading**

In future, the authors may benefit from using an English language copy editor and proof-reader before submitting their manuscript. This will spare the reviewers much work! Please do a final English language proofread of the document to improve readability. Some grammatical and copy-editing errors that I detected include:

Introduction:

- “One of the most challenging part of…” should say “parts”
- “even very pandemic- or COVID-19-specific challenges” – not clear what is meant here by “even very” – suggest to remove “very” and clarify
- “Based on the our own survey results [2],” remove either “the”
- “Based on the our own survey results [2]… led to two topics that are of particular practical and ethical relevance:” – grammar wrong, please rephrase eg, “… two two topics that are of particular practical and ethical relevance were found:”
- “to investigate pandemic-specific prevention or therapy approaches” – therapeutic approaches
- “but too little patients are eligible” – few patients
- “*informed consent* and *study prioritization in a* pandemic setting” - in a should not be in italics

Methods:

- “of research ethics committees, of regulatory bodies, of public sponsors, of guardianship courts and authorities, and.” Correct to: “of research ethics committees, regulatory bodies, public sponsors, guardianship courts and authorities.”
- “The recruitment of interviewees followed a purposive sampling approach..” remove additional period
- “until a consensus was reached.” Remove “a”

Results:

- “can be found in supplementary table S2” – sentence lacks a period at the end
- When adding direct quotes within a sentence (or anywhere in the manuscript really), please use “ ” rather than ‘ ’. Please change throughout manuscript.
- “decision making competence” – should read decision making capacity
- “relevance for interventional studies’ (C15).” – should read intervention study, not interventional (please check rest of manuscript for this errors, as I also saw it pop up in a few other places)
- “… routine data and biomaterials (C17).” Biomaterials is not a commonly used term, I think what you mean is biological samples? (change throughout manuscript)
- “Secondary-use of patient data” – there should be no hyphen (-) after secondary
- “asked from different physicians at the same time to participate in their clinical trials (P2).” – should say “asked by different physicians…”
- “As above mentioned, in some occasions one patient was asked to participate in several studies illustrates the benefit of…” – should say “as mentioned above” and “illustrating”
- “While discussing the issue of prioritization different interviewees mentioned” – add a comma after prioritization

Discussion

- “The more the validity conditions for informed consent are challenged the less does the consent procedure serves” – serve not serves
- Recovery Study conducted in the UK [10] - RECOVERY should be capitalized
- “To be emphasized is the IC agreement,” – please be consistent with use of acronyms vs spelling out in full. This seems to be the first use of IC and the acroym is not explained
- “Prioritization aspects: When reflecting the urgent need to” – should read “when reflecting on the”
- “Because prioritization of specific studies can not only be helpful for scientists by improving quality but also protect patients by increasing the chance that primarily robust and clinically highly relevant studies are initiated.” – remove because at the beginning of the sentence
- “Prioritization and collaboration efforts to improve the scientific justification of trials conducted in the pandemic context would this function as an important compensation for the above mentioned challenges with the informed consent procedure.” – sentence doesn’t make sense, do the authors mean “thus” instead of “this”?

**References**

- Please get this in order, not all references are properly formatted yet

**Data availability statement**

While I agree that data should not be made publicly available because of risk of re-identification of participants. However, in line with PLOS One guideline, it would be advisable for the authors to consider how they will deal with secondary requests for data. For example, they may wish to state that upon reasonable request, a list of condensed meaning units or codes from the interview transcripts can be made available on request to the corresponding author/data sharing committee of XX university (as appropriate).
