# Supplementary material for: Informed consent and trial prioritization for clinical studies during the COVID-19 pandemic. Stakeholder experiences and viewpoints
Source: PLoS One. 2024 Apr 30;19(4):e0302755. doi: 10.1371/journal.pone.0302755 (PMC11060594; doi:10.1371/journal.pone.0302755)
Supplement: Attachment — Submitted filename: Responses to reviewer comments final.docx [file pone.0302755.s004.docx]

Authors’ responses to reviewer comments

General comment from the authors: We thank both reviewers and the editor for their time invested and for their many very thoughtful comments that in our opinion helped to improve the manuscript. In the following, we respond to each comment and highlight what we changed in the manuscript. We further provide the clean version of the revised manuscript and another version with tracked changes. We also substantially revised the wording in the supplementary table 2 and thus also attach a version with tracked changes for this table.

Reviewer #1:

Reviewer comment: This paper addresses an important, interesting, and timely topic. The chosen methods of qualitative research seem appropriate to dig deeper into some preliminary topics identified in a prior literature review conducted by the authors. However, the methods and results section would benefit from further details, while it is also advised that the authors engage more substantively with existing literature in the discussion section. Detailed comments for each section of the paper are provided below. I hope will help to strengthen the impact of this timely paper.

**Introduction:**

“Valid informed consent exists only if the patients concerned were a) competent to make decisions in the situation in question, b) received the relevant information, c) understood it, and d) were free from coercion to decide for or against consent.” Please add a relevant reference to research ethics guidelines (eg. CIOMS, Helsinki) or seminal literature.

Authors’ response: We added references accordingly

Reviewer comment: “Prioritization of clinical studies that is the ranking of more or less important trials might become relevant, for example, due to a shortage of eligible patients.” The strain/shortage of human resources in the health care sector could be and additional important factor to name here.

Authors’ response: We agree with the reviewer and added this information accordingly. The new text is:

“Prioritization of clinical studies that is the ranking of more or less important trials might become relevant, for example, due to a shortage of eligible patients or available investigators and trial nurses for conducting trials.”

Reviewer comment: “Recent studies showed that many clinical trials, for example in Germany, failed due to insufficient recruitment [5]. Prioritization might also be important if too many studies are conducted in an inefficient or even harmful way. For example, studies showed that many trials investigated the same therapeutic approach at the same time [6].” It seems reference 5 is a literature review of some sort, so say this in text. What kind of study is reference 6 referring to? It would be good to briefly add some context to both these references in text e.g., where these study/these studies were conducted and what they investigated.

Authors’ response: We agree that these references need more context. We added relevant text to the manuscript. The respective text is:

“A recent review of registered trials showed that many clinical trials, for example in Germany, failed due to insufficient recruitment [7]. Prioritization might also be important if too many studies are conducted in an inefficient or even harmful way. An explorative review or registered trials showed that many trials investigated the same therapeutic approach at the same time [8].”

Reviewer comment: “The results of the interview study shall inform the development of practice oriented guidance for informed consent and trial prioritization as a component of pandemic preparedness.” Can you strengthen this point/argument by specifying which part of pandemic preparedness are you talking about.

Authors’ response: We added relevant text. The revised text is:

“The results of the interview study shall inform the development of practice-oriented guidance for informed consent and trial prioritization as a component of pandemic preparedness activities. Respective national activities are, for example, the German PREPARED (Prepardness and Pandemic Response in Deutschland) but also trials-focused network activities such as the German NUKLEUS platform for developing standards to plan, conduct and evaluate large-scale clinical and epidemiological studies nationwide.”

**Methods:**

Reviewer comment: “Based on typical sample sizes in qualitative research and own experiences we estimated that 15-20 interviews are needed to reach thematic saturation on the level of major and first-level subgroup categories.” It seems to me that this sentence reflects more of a quantitative paradigm than a qualitative one. In qualitative research there is no such thing as a ‘typical sample size’ and it is neither necessary nor advisable to state estimated sample sizes, especially when seeking to obtain thematic saturation. Data collection and analysis should be conducted iteratively, continued until saturation is reached. If the authors (for research or time constraints) chose to pre-empt their sample size, estimating it to be 15-20 interviews (as stated here), it should not be claimed the study also adhered to principles of thematic saturation, as this is a contradictory claim. If the former was the case, please remove references to thematic saturation and describe in the limitations section that only this was a rapid qualitative study, in which only 21 interviews were conducted because of time and resource constraints. Particularly given the wide range of stakeholder groups included in this study (clinical investigators, clinicians from clinical trial centers, clinic directors, patient representatives, members of research ethics committees, of regulatory bodies, of public sponsors, of guardianship courts and authorities”) it seems very unlikely that saturation could have been predicted to be reached at 15-20 interviews.

Authors’ response: We fully agree with the reviewer. Sample size estimates are not meaningful in qualitative research. The number of 21 interviews signals that we did not aim to reach the mentioned estimates but thematic saturation. We deleted the relevant sentence. The remaining text signals that we purposively sampled stakeholders until we reached thematic saturation of the major and first-subgroup level for categories.

Reviewer comment: “normal ward” is not a widely used or accepted term. Please specify which kind of ward you are referring to when you say ‘normal’

Authors’ response: We changed the text to “non-intensive care wards including internal medicine”

Reviewer comment: “We further identified potential interview participants via snowballing.” Please give more detail on who this snowball sample originated from? Was this from those participants already recruited purposively via the QUEST Center, AKEK and PRECOPE?

Authors’ response: Yes, we asked during the interview if participants could recommend someone to be included in this study. Several of these interview partners were initially identified via recommendations from the PRECOPE advisory board that included representatives from relevant umbrella organizations for clinical research, health care, and patient organizations. We clarified this in the publication.The relevant text is:

“The recruitment of interviewees followed a purposive sampling approach. We identified representatives of the above mentioned stakeholder groups via the network of the QUEST Center, the AKEK (German working group of research ethics committees), and the PRECOPE advisory board that included representatives from umbrella organizations for clinical research, health care, and patient organizations. We further identified potential interview participants via snowballing using recommendations from interviewees.”

Reviewer comment: “We conducted the interviews via videoconferencing…” please specify which videoconferencing platform was used. Please also state somewhere here that interviews were recorded (say if audio or video recorded), as this is currently not explicitly stated (you only mention that they were transcribed).

Authors’ response: We added the platform (MS Teams). We also added the following text:

“All interviews were video recorded and saved as an audio file. A transcription company transcribed the audio file under a confidentiality agreement. MAXQDA 2020 was used to process and manage the interviews and enter coding results.”

Reviewer comment: “MAXQDA 2020 was used for the analysis of the interview material.” Please clarify that the software was used to process and manage the analysis, as the analysis itself cannot be done by the software.

Authors’ response: We thank the reviewer for this comment. We changed the wording as of course we used the software only to conduct the analysis.

Reviewer comment: “We used qualitative content analysis according to Mayring (2010) [9].” Please add a sentence or two which summarizes the key components of QCA by Mayring for readers not familiar with this approach.

Authors’ response: We added the following text:

“Qualitative Content Analysis (QCA) according to Mayring involves categorizing the interview content into predefined categories, ensuring reliability and validity through a controlled process, and then interpreting the results in the context of the research question”.

Reviewer comment: The data analysis section is rather brief and would also benefit from further details:

- on the research team and reflexivity (including the each of the researcher’s background and positionality and how this may have impacted participant’s responses in terms of social desirability, openness etc.; why the choice was made to have an observer present during interviews and how may this have impacted the interviewer-interviewee dynamics)

Authors’ response: We added the following text to the methods section:

“The research team comprised of individuals with expertise in medicine, public health, and bioethics. The team did not have personal acquaintances with the interview partners. The decision to include an observer during interviews was made to foster reflective discussions on the interview responses.”

Reviewer comment: It is also notable that three authors, plus one observer each, collected the data but one author did the bulk of analysis. Please provide more details on the following questions:

- Why was this approach chosen rather than analysis within the wider research team?
- What was done with the notes taken by the observers present during interviews? Did these feed into analysis and if so how? If not, what function did the notes serve?
- How did the research team community among themselves to share emerging findings during data collection?
- Was analysis done iteratively or after all data had been collected? If so, how did this impact assessment of saturation, which the authors claim guided them in deciding how many interviews to conduct.

(Later on when reading the author contributions I noted that it says there “SW, AF, DS and LW analyzed and interpreted the data”, please be consistent and clarify how analysis was conducted.)

Authors’ response: We revised the Data Analysis part and thereby addressed the helpful questions of the reviewer. The revised section is:

“Data analysis: We employed qualitative content analysis according to Mayring (2010) [11]. This method involves categorizing the interview content into predefined categories, ensuring reliability and validity through a controlled process, and then interpreting the results in the context of the research question. The analysis was characterized by an inductive development of themes. The transcripts were initially coded by SW. To ensure accuracy and consistency, AF verified the coding on the first 10 transcripts. Coding commenced immediately upon receipt of the first transcript. Notes from observers helped to interpret the transcripts and were available for the whole research team. This iterative coding approach informed our purposive sampling strategy for subsequent interview participants and helped us ascertain when we had achieved thematic saturation. LW provided oversight and actively participated in the early stages of the coding process. All codes and discrepancies within the coding were discussed and further modified in an iterative process in several group meetings as well as written feedback rounds within the research team (SW, LW, AF, SGS, DS) until consensus was reached. The entire analysis was conducted in German. For the purpose of this paper, relevant quotes were translated into English.”

**Results:**

Reviewer comment: “thematic (qualitative) saturation” – please remove the word qualitative, the term is thematic saturation

Authors’ response: We deleted “(qualitative)”.

Reviewer comment: “Altogether, we contacted 51 stakeholders, which gives a response rate of 42%.” Again this sounds very quantatative. In qualitative research we are not really concerned with things like response rate, as it doesn’t really tell us anything at all. Instread, I would suggest simply saying that you contacted and invited 51 people to take part in the study. In case you collected reasons why prospective participants chose not to participate, then you could add these here, as that would be interesting, rather than giving a response rate.

Authors’ response: We changed the sentences accordingly. We did not collect reasons why people declined to take part in the interview. We provided the number of invited people to illustrate how often our initial purposive sampling resulted in interviews.

Reviewer comment: “For the first topic informed consent we identified three main categories” – is there a reason why you refer to categories rather than themes?

Authors’ response: We thank the reviewer for pointing to this inconsistency. We changed it to themes to be consistent with “thematic saturation”

Reviewer comment: “For the first topic informed consent we identified three main categories: consent challenges, impact of consent challenges on clinical research, and potential response strategies for consent challenges. For the second topic prioritization of trials, we identified two main categories: need for prioritization of clinical studies, potential response strategies for prioritization of clinical studies.”

– suggestion to remove “for prioritization of clinical studies” as this is quite lengthy for a theme heading and to be consistent with the theme heading under the topic of ‘informed consent’

Authors’ response: We changed the themes to “potential strategies for consent challenges” and “potential strategies for prioritization” as suggested below.

Reviewer comment: “first and second level subcategories” – again, why are these not called subthemes?

Authors’ response: Thanks. We changed it to subthemes as well.

Reviewer comment: S2: there are some inconsistencies in the details of reported quotes, with some of them stating “(Interview X)” others “(Interview X, pos. XX)” or “(Interview X, item XX)”. Please ensure there is consistency across the table. If using posX or item X, please explain in a footnote to the table what this is referring to, as it’s not clear to the reader and not a standard way of reporting quotes. Usually, some non-identifiable details of participants (e.g. stakeholder category, gender etc.) are stated after the Interview X, … to give the reader an idea of the positionality of the participant sharing this view.

Authors’ response: We deleted the specifications, like “pos.” or “item”. They were rather for internal use. We decided not to provide further characteristics of the interviewees as they could be re-identified.

Reviewer comment: I like the authors use of direct quotes in text throughout the results section. However, the manuscript would benefit from several full quotes, which exemplify the point being made, from being added in text to the themes being discussed. This would enrich the results section and give readers who do not prioritise consulting table S2 in detail (likely to be most readers!) a flavour of the data and the kinds of views being expressed by participants. I suggest that you add 2-3 key quotes within each of the broad level themes (i.e. 2-3 quotes under Consent challenges, Impact of consent challenges on clinical research and so on…).

Authors’ response: We fully agree. We added 2-3 quotes for each broad theme and believe that this helps the reader to get a better understanding from both the flavour of data and also the process of thematic analysis and coding.

Reviewer comment: The theme of “Isolation and hygiene conditions” seems to also describe conditions of physical distancing more generally, not just isolation. Consider revising title of this theme to reflect that e.g. “Isolation, physical distancing and hygiene conditions”

Authors’ response: We agree with the reviewer. Many thanks again. We changed the theme accordingly.

Reviewer comment: The theme of “Overburdening the patient” – “Patients often had difficulties in ‘understanding due to complexity’ of informed consent documents. This was further complicated by the pandemic context (C8).” Why and how was this further complicated by the pandemic context? Please explain. (e.g., This is one example of where adding a full quote could help to enrich results and make them more understandable).

Authors’ response: We included a quote in the main text to illustrate that the “overburdening” consisted of information overload but also the fear of participation in a study on a disease that is not fully understood (the “pandemic context”).

Quote (interview 6): *“For example, doctor A goes to the patient and asks: "Would you like to take part in the XY drug trial? The patient asks for time to think about it. During the reflection period, Doctor B comes and clarifies for the next study. And the patient thinks it's the same trial and says yes. These things can happen.”*

Reviewer comment: Under “Impact of consent challenges on clinical research”

“The challenges around appropriate consent procedures for COVID-19 trials had an impact on conducting clinical research:” – please be careful how you phrase results, as you are not stating facts here but rather reporting on participant’s perceptions of a particular situation. Suggestion to rephrase as something like “Many/some/all interviewees reported that the challenges around appropriate consent procedures for COVID-19 trials had an impact on conducting clinical research”. (similarly with the sentence “‘Sustainable funding’ (P24) becomes important to make the prioritization procedures effective.” later on in the results section – please rephrase so its clear that this represents participants views)

Authors’ response: We thank the reviewer for this right distinction between generalizable issues (“facts”) and perceptions of interview participants. We rephrased these sentences.

Reviewer comment: Under “Opt-in consent as ethical standard”: “…some interviewees addressed the protective function of the informed consent for potential ‘conflicts of interest’ of clinician investigators (C16).” Please add more explanation or a quote here, as it’s not clear from this sentence what is meant here.

Authors’ response: We included a quote in the main text.

Quote (interview 3): *“Never [change consent in times of crisis]! Doctors always have conflicts of interest.”*

Reviewer comment: Theme “Secondary-use of patient data”: this theme contrasts nicely with the “Opt-in consent as ethical standard”, suggesting some difference in views when it comes to intervention studies vs studies of secondary/routine data. Suggestion to adjust the theme heading to make this difference clearer, e.g. by calling it “Opt-out consent for secondary-use of patient data”

Authors’ response: We really like the idea of contrasting “opt-in consent as ethical standard” with “opt-out models in secondary use of patient data”. Unfortunately, “opt-out in secondary use of patient data” does not cover all subthemes. Interviewees also referred to the model of broad consent and to the use of aggregated routine data without any consent. We nevertheless decided to rename the categories to “Opt-in consent as ethical standard for interventional studies” and “Consent models for secondary use of patient data”.

Reviewer comment: Theme “Patients incapable to give consent”: please rethink the title of this theme, as it isn’t very meaningful when considered considering the top-level theme for this category of results, which is about “Potential response strategies for consent challenges”. The title of the theme should be self-explanatory with regards to what the response strategy here is (e.g. opt-in or opt-out), while “Patients incapable to give consent” is not a response strategy.

Authors’ response: We fully agree and changed the theme to “Requirements for patients unable to give consent”

Reviewer comment: Theme “Need of prioritization” – it seems to me that this theme is rather describing the “perceived benefits of prioritization”. Consider renaming to make this more self-explanatory. If so, I would also suggest renaming subheadings such as “ensuring patient protection” “improving validity and efficiency” “increasing relevance of trials”. In general, it’s desirable for theme headings to be able to stand alone and make sense on their own, even before reading the detailed results.

Authors’ response: Again, very good point. We renamed the theme and subthemes accordingly.

Reviewer comment: Theme: “Lack of explicit prioritization” consider moving this to be right at the top of the section, followed by the other three themes. I think that order would flow more naturally, giving context to the other three themes which focus on the perceived benefits of prioritization.

Authors’ response: We agree and changed the order accordingly.

Reviewer comment: Theme: “Potential response strategies for prioritization” consider simplifying title to “Potential strategies for prioritization”, as the word response doesn’t add much but makes title unnecessary technical sounding

Authors’ response: We changed the name of the theme.

Reviewer comment: Theme: “Study design” similar to my above comment about making themes meaningful and self-explanatory, I recommend renaming this into something like “adapting study design”. This theme doesn’t appear very rich. Is there any interesting data the authors could to add to this theme?

Authors’ response: We renamed the subtheme and added quotes and some more explanation to make this section richer in content.

**Discussion:**

Reviewer comment: Section discussing “Informed consent” – there is a great wealth of research ethics literature that has engaged with the question of how to achieve informed consent in research generally and in emergency situations more specifically. I would like to see the discussion on the findings around informed consent engage with some of this literature, in light of the perceptions of the stakeholders shared. For example, what are some of the methods that have been developed in other contexts which could also help to serve informed consent in this setting? A discussion of some of the literature on community engagement and its role in achieving informed consent, as well as on ‘broad consent’ models also seems important here. As it stands, the discussion does not seem sufficiently embedded and engaged with the literature and academic debate on this topic. The authors have done a nice job when discussing the criteria of Meyer et al. [8] in section that followings and should similarly engage with the literature in the discussion on informed consent.

Authors’ response: We agree that much more could be said to contextualize our results into the great wealth of literature on informed consent. The initial text already contextualized our results by referring to international activities for adapted consent models within the COVID-19 pandemic such as in LMIC and in Great Britain. We revised the whole section and added more discussion, context, and references to the broad consent part and to the issue of deferred consent.

**Limitations:**

Reviewer comment: Please expand on the first limitation to explain how this limited findings, given you also say it was justified by the literature review.

Authors’ response: We revised the text as follows:

“Firstly, the content focus on informed consent and study prioritization was set by us. As mentioned in the introduction, our focus on these two topics was justified by literature-based preliminary work, a survey study across German research ethics committees, and consecutive expert consultation. Other research ethics issues such as transparency of research results were partly addressed by other activities from our group [24] but the need for a better qualitative understanding of SWOT was judged by external experts and our group to be highest for the two issues we focused on”

**General comments:**

Reviewer comment: In future, please use line numbers on your manuscript submissions, so that reviewers can refer more easily to different sections of the paper, rather than having to copy and paste each sentence. This will make life easier for the reviewer and also for the authors when addressing reviewers comments.

Authors’ response: We apologize for not using line numbers. Again, we thank the reviewer for the very thoughtful and helpful comments and the surely time-consuming work he/she has put into this review.

Reviewer comment: Please follow PLOS ONE style guidelines with regard to second level headings. Currently some second level headings are not clearly visible, for example in the methods section.

Authors’ response: Many thanks, we added second-level headings to the Methods section.

Reviewer comment: S1- topic guide: in places this reads more like a survey, rather than an in-depth interview. For example: “Have you come into contact with this issue at your center?” If yes: check here regarding decisions between studies that were ready in parallel to the initiation and regarding studies that were recruited preferentially” – it is unclear to me what needs to be “checked” here and by whom? Please clarify and correct if this is a typo

- Suggestion for authors to use more open ended questions in future qualitative studies, as these generally yield richer and more unexpected data.

Authors’ response: All bullet points of the interview guide were only optional topics to support those conducting the interviews. We primarily addressed open questions on the first-level questions and topics. We agree that the specific sentence you mentioned is unclear. Because we also did not addressed this specific question in our interviews we deleted it from the supplement file to avoid misunderstanding.

Reviewer comment: Study lacks the perspective of patients. It would have been interesting to hear from them on their views on trial participation and consent, as surly they are an important ‘stakeholder’ in this topic. The authors mention this in the limitations section but do not give a clear reason for why patients were not included. Please discuss this further in limitations section.

Authors’ response: We expanded the discussion section to explain this limitation further. We tried to identify interview partners that can represent the patient experiences beyond their individual experience but also the patient organizations that we contacted and the member of our advisory board who is representing a big national patient organization could not identify such a person. It seems as if patient organizations did not have internal discussions on informed consent experiences in COVID-19 trials. We believe however, that a stand-alone interview study with a purposive sample of 15-20 patients that were included in COVID-19 trials will be of value. We mention this in the revised discussion section as follows:

“Secondly, while we were able to include diverse perspectives from trialists, health care professionals, regulatory bodies and despite our contact with patient organizations we were not able to identify patient representatives that could comment more generally on experiences with informed consent and study prioritization in the pandemic context. We believe that both a qualitative interview study and a quantitative survey of trial participants and/or their legal representatives would be an important follow-up work on our explorative qualitative expert interviews”

Reviewer comment: Different stakeholders’ perspectives – I was surprised that the authors did not discuss anywhere in the results and discussion the similarities and differences in views among different stakeholders included in this study. This leaves me wondering why the authors chose to recruite a variation sample if then failing not to compare and contrasting the views of different stakeholders? It is assumed that all stakeholders share the same views? This seems highly unlikely. One characteristic of rich qualitative research is that it can bring out nuanced views and explain them in light of the positionality/perspective of who has shared those views, as well as being able to shed light on deviant findings. Please

could the authors respond to this and clarify why different stakeholders’ perspectives are not discussed, or if relevant to revise the results and discussion section accordingly.

Authors’ response: We thank the reviewer for this important comment and we can understand that the reviewer was surprised about not reading more about differences across stakeholder groups. When reflecting on the reviewer’s comment we realized that indeed the different interview responses did not reveal differences as a core result. This might have to do with the fact that for both topics, informed consent and prioritization during the pandemic, interview participants primarily described challenges and potential mitigation strategies in more general terms. We believe that this very explorative level of information is important as a first step. These perspectives however did not result into, for example, conflicting positions on challenges but rather into a broad set of different challenges or mitigation strategies. The goal of our interviews was not to go into details regarding the practical implementation of solution strategies. We believe that positionality and differences across stakeholder feedback would become much more prominent for an interviews study focusing on such implementation questions. We added further content to the discussion to make this point more explicit. The relevant text is:

“A reason for why our interview results do not contain core differences in stakeholder feedback might be that our interviews did not address details for how to implement mitigation strategies, We believe that positionality and differences across stakeholder feedback would become much more prominent for an interviews study focusing on such implementation questions.”

And later

“The results of the interview study shall inform the development of practice-oriented guidance for informed consent and trial prioritization as a component of pandemic preparedness. As mentioned above, further patient and stakeholder engagement would be key in the early identification of different stakeholder perspectives on most suitable mitigation strategies.”

**Proofreading**

Reviewer comment: Reviewer comment: In future, the authors may benefit from using an English language copy editor and proof-reader before submitting their manuscript. This will spare the reviewers much work! Please do a final English language proofread of the document to improve readability. Some grammatical and copy-editing errors that I detected include:

Introduction:

- “One of the most challenging part of…” should say “parts”
- “even very pandemic- or COVID-19-specific challenges” – not clear what is meant here by “even very” – suggest to remove “very” and clarify
- “Based on the our own survey results [2],” remove either “the”
- “Based on the our own survey results [2]… led to two topics that are of particular practical and ethical relevance:” – grammar wrong, please rephrase eg, “… two two topics that are of particular practical and ethical relevance were found:”
- “to investigate pandemic-specific prevention or therapy approaches” – therapeutic approaches
- “but too little patients are eligible” – few patients
- “*informed consent* and *study prioritization in a* pandemic setting” - in a should not be in italics

Methods:

- “of research ethics committees, of regulatory bodies, of public sponsors, of guardianship courts and authorities, and.” Correct to: “of research ethics committees, regulatory bodies, public sponsors, guardianship courts and authorities.”
- “The recruitment of interviewees followed a purposive sampling approach..” remove additional period
- “until a consensus was reached.” Remove “a”

Results:

- “can be found in supplementary table S2” – sentence lacks a period at the end
- When adding direct quotes within a sentence (or anywhere in the manuscript really), please use “ ” rather than ‘ ’. Please change throughout manuscript.
- “decision making competence” – should read decision making capacity
- “relevance for interventional studies’ (C15).” – should read intervention study, not interventional (please check rest of manuscript for this errors, as I also saw it pop up in a few other places)
- “… routine data and biomaterials (C17).” Biomaterials is not a commonly used term, I think what you mean is biological samples? (change throughout manuscript)
- “Secondary-use of patient data” – there should be no hyphen (-) after secondary
- “asked from different physicians at the same time to participate in their clinical trials (P2).” – should say “asked by different physicians…”
- “As above mentioned, in some occasions one patient was asked to participate in several studies illustrates the benefit of…” – should say “as mentioned above” and “illustrating”
- “While discussing the issue of prioritization different interviewees mentioned” – add a comma after prioritization

Discussion

- “The more the validity conditions for informed consent are challenged the less does the consent procedure serves” – serve not serves
- Recovery Study conducted in the UK [10] - RECOVERY should be capitalized
- “To be emphasized is the IC agreement,” – please be consistent with use of acronyms vs spelling out in full. This seems to be the first use of IC and the acroym is not explained
- “Prioritization aspects: When reflecting the urgent need to” – should read “when reflecting on the”
- “Because prioritization of specific studies can not only be helpful for scientists by improving quality but also protect patients by increasing the chance that primarily robust and clinically highly relevant studies are initiated.” – remove because at the beginning of the sentence
- “Prioritization and collaboration efforts to improve the scientific justification of trials conducted in the pandemic context would this function as an important compensation for the above mentioned challenges with the informed consent procedure.” – sentence doesn’t make sense, do the authors mean “thus” instead of “this”?

Authors’ response: Thanks a lot for carefully reading the manuscript. We adapted the vast majority of the points raised. We did a proof reading of the entire manuscript again and we revised accordingly. The only recommendation that we did not follow is to change interventional studies in intervention studies. Both terms are established with “interventional” being even more established in formal texts. Check for example the glossary of the most employed registry clinicaltrials.gov that list interventional study but not intervention study. Also the EU laws and the EMA mainly speak of “interventional” and “non-interventional” studies. https://www.ema.europa.eu/en/human-regulatory/research-development/clinical-trials-human-medicines

**References**

- Please get this in order, not all references are properly formatted yet

Authors’ response: We are using Endnote as reference manager. After our update of the reference list we checked all formatting but did not find errors in this updated version.

**Data availability statement**

Reviewer comment: While I agree that data should not be made publicly available because of risk of re-identification of participants. However, in line with PLOS One guideline, it would be advisable for the authors to consider how they will deal with secondary requests for data. For example, they may wish to state that upon reasonable request, a list of condensed meaning units or codes from the interview transcripts can be made available on request to the corresponding author/data sharing committee of XX university (as appropriate).

Authors’ response: We thank the reviewer for this comment. We checked the informed consent text again and believe that the wording strongly limits data sharing in this case. We are already in a process with our local data protection officers to discuss appropriate wording in the consent form that would in the future allow data sharing including upload of pseudonymized data in repositories. In the case of this paper we added a sentence as suggested. The text is:

“a list of pseudonymized meaning units from the interview transcripts in addition to material provided in the supplementary table 2 can be made available on request to the corresponding author”

Reviewer #2: Thank you for the opportunity to review this manuscript. I enjoyed reading it and the issue addressed - informed consent – is one faced by all researchers, and one which becomes more complex in clinical trials taking place in a rapidly shifting pandemic. The response strategies provided by the authors are practical, feasible and drawn from interview data collected from experts with direct experience in relevant fields.

Reviewer comment: My overall feeling, however, is that there is the potential for much deeper reflection - I was left wanting more detail from the results. I would like the authors to situate their findings within a wider explanation of why informed consent is important for potential research participants as well as institutions and researchers. The authors note the lack of participant voices as a limitation of the study, but I would still like to see more discussion of the informed consent process as a protective mechanism as well as one which advances scientific knowledge. This would help to balance out the arguments and frame the findings in a more patient-centred way, placing the protection of vulnerable patients (or their data) at the centre and stressing the importance of a thorough and ethical informed consent process around them to improve future health interventions/treatment.

Authors’ response: We agree with the reviewer and in line with comments from the other reviewer we added several details to the results part. Furthermore, we added context at several parts in the Background, Results, and Discussion section to put the two topics “informed consent” and “study prioritization” into context. In the Background section for example we highlight that the “informed consent process shall allow eligible patients to make their own judgments about benefits, risks/burdens, and other relevant aspects that come with study participation. Based on these judgements the patients make informed decisions about whether to participate or not. Valid informed consent exists only if the patients concerned were a) competent to make decisions in the situation in question, b) received the relevant information, c) understood it, and d) were free from coercion to decide for or against consent [3, 4]. Critical care settings in general bear several challenges for ensuring a valid consent process due to difficult patient- and context-related circumstances that decrease decision making capacity [5, 6]. A pandemic situation with social distancing measures in place and high uncertainty regarding potentially effective treatments can further aggravate these challenges. (...) At the same time the efficient and effective recruitment of eligible patients for clinical studies to investigate pandemic-specific prevention or therapeutic approaches is of utmost importance..

Reviewer comment: I have two main comments before addressing other minor issues below:

1. I feel that the authors are actually addressing two different issues in one: informed consent and trial prioritisation. This may not be in line with other reviewers, but I wonder if it would be beneficial to divide this into two separate manuscripts or if not, focus on informed consent with prioritisation discussed as one theme/solution. This would give the authors more space to present their data in detail and make it a stronger qualitative piece of work.

Authors’ response: We appreciate your suggestion to divide the paper into two separate manuscripts or focus on informed consent with prioritization discussed as one theme/solution. However, we believe that keeping the two topics (informed consent and study prioritization) in the same manuscript is more beneficial for the following reasons:

Interconnectedness of the topics: Our paper demonstrates and discusses the linkage between informed consent and trial prioritization. The Discussion section highlights that mitigation strategies for challenges with informed consent and study prioritization partly share common ground, as high-quality procedures for study prioritization seem to be a core mitigation strategy in dealing with informed consent challenges. By addressing both topics in a single manuscript, we can better illustrate how the challenges and mitigation strategies for informed consent are connected to study prioritization.

Practical importance of both topics: Both informed consent and trial prioritization were selected as practically important topics by the consulted stakeholder groups. By including both topics in the same manuscript, we can provide a comprehensive view of the challenges and mitigation strategies faced by stakeholders involved in clinical research during the COVID-19 pandemic.

Reviewer comment: 2. The way the qualitative data is presented means the substance and detail of it is hidden: quotes and examples are lost and over-summarised. The manuscript would benefit from the results being integrated into the main text. I recognise that word count may be an issue, but most of the sections in the results section are very short and need to be expanded to provide more detail and context.

Authors’ response: In line with comments from the other reviewer we integrated more 2-3 quotes for each broad theme in the text in order to give a deeper insight into the answers of the interviewee.

Introduction/abstract
Reviewer comment: Could the authors provide a definition or explanation of ‘practice-oriented challenges’?

Authors’ response: We deleted the word “practice-oriented challenges” and wrote more descriptively “how these challenges (…) have played out in practice

Reviewer comment: Whilst I recognise that ‘human subject’ is a common phrase in clinical trial literature, it removes some of the agency of the research participants by reducing them to ‘subjects’. Would it be possible to reword the title or offer an explanation/recognition that the use of the word ‘subject’ can be complicated? Language such as being ‘incapable’ of consenting should also be amended.

Authors’ response: We changed the title and use now “clinical studies” and at other part in the text we changed to “clinical research”. We also do not use the “incapable” anymore.

Reviewer comment: Can you provide more epidemiological background on the Covid context in Germany at the time of the study? A few sentences here would help the reader to situate the findings and understand the context in which the key informants were working.

Authors’ response: We added the following text to the background section:

“From the beginning of the COVID-19 pandemic until July 2021, Germany experienced several waves of infections with daily new cases reaching over 30,000 in winter 2020. In response, the German government implemented strict lockdown measures, including closures of non-essential businesses, schools, and public spaces. As the vaccination campaign progressed in 2021, the number of daily new cases began to decline, and restrictions were gradually eased. By July 2021, the country was in a phase of reopening.“

Reviewer comment: I would also have liked to see more examples of why informed consent and ethics are important in clinical trials, for example, through citing ethics guidelines or describing what the informed consent process means for individual research participants, not just the researchers using their data.

Authors’ response: We agree that it is important to stress out why informed consent is important in clinical trials and what it means for patients. In the Background section we extended the text that introduces the importance of informed consent and we cite (as we did in the initial version) the Declaration of Helsinki and CIOMS:

“The informed consent process shall allow eligible patients to make their own judgments about benefits, risks/burdens, and other relevant aspects that come with study participation. Based on these judgements the patients make informed decisions about whether to participate or not. Valid informed consent exists only if the patients concerned were a) competent to make decisions in the situation in question, b) received the relevant information, c) understood it, and d) were free from coercion to decide for or against consent [3, 4]. Critical care settings in general bear several challenges for ensuring a valid consent process due to difficult patient- and context-related circumstances that decrease decision making capacity [5, 6]. A pandemic situation with social distancing measures in place and high uncertainty regarding potentially effective treatments can further aggravate these challenges“.

Methods
Reviewer comment: The methods are well described and appropriate for a study of this kind, which relies on key informant interviews of a technical nature.

Reviewer comment: Could the authors be consistent in how the study is described? I would be tempted to call it a ‘qualitative research study’ which draws on data from key informant interviews, rather than a qualitative interview study.

Authors’ response: We changed the description of our study depending on the context in the manuscript to “interview study” or “qualitative study”

Reviewer comment: Thank you to the authors for being honest about the response rate – this is typical of this kind of research, in which the interviewees are stakeholders in busy academic, coordination and research positions. I would move the response rate into the methods section, and link it to the description of your sampling process.

Authors’ response: Because the other reviewer recommended to completely remove the response rate we followed the advice but kept the numbers of invited people to illustrate how often our initial purposive sampling resulted in interviews.

Reviewer comment: Would the sub-heading ‘procedure’ be better described as ‘data collection’?

Authors’ response: We thank the reviewer for this suggestions and changed the text to “data collection”.

Reviewer comment: What language were the interviews conducted in, and was any translation required at the same time as transcription (eg German to English)?

Authors’ response: Interviews were conducted, transcribed, and analysed in German. Quotes were translated for this publication. We added this explanation in the manuscript.

Reviewer comment: The ethical-legal analysis mentioned in the introductory comments of the interview guide is not specifically mentioned in the methods – was this part of the study design? If so, could you mention it in the methods?

Authors’ response: No, the ethical-legal analysis is another task that is informed by the interview results but rather happened after the interview study. We only wanted to inform the interview participants about the scope of our research interests.

Results section
Reviewer comment: I recognise that the results section is a presentation of the data which is discussed and later reflected upon in the discussion, but it is very short and would benefit from the inclusion of more examples.

Authors’ response: See our comment above. We included much more quotes in the revised Results section.

Reviewer comment: Upon reading the manuscript for the first time I assumed that C3, C4 etc were individual interviewee codes, used to prevent key informants from being identified, rather than referring to a theme linked to specific quotes. I would like to see the quotations integrated into the main body of the paper, not only in the table - the table is lengthy and my concern is that readers will not go back and forth to search for the examples, meaning that the impact of the qualitative nature of the data is lost.

Authors’ response: See also above. We included more specific quotes in the text.

Reviewer comment: I suggest restructuring the results so that each theme is introduced in more detail (eg it jumps from a heading of informed consent, to headings on consent challenges and time pressure without any description of the content to follow). I would like to see more ‘linking’ sentences such as ‘many of the interviewees described the time pressures they faced in obtaining consent…’ or ‘this was raised as an issue by many interviewees…’

Authors’ response: When revising the Results section and by introducing more quotes we also added further linking sentences between technical terms for “themes” and the added quotes for example.

Reviewer comment: Some of the results sections only include one sentence – this makes the manuscript harder to read and suggests that there may not be enough data in some of the categories to describe them in detail. Can you merge some of these sub-headings, or develop them more so that they are not just one phrase or sentence?

Authors’ response: See our responses above. We believe that the many additional quotes and further revisions to the Results section improved its readability and information value.

Reviewer comment: This sentence is not clear to me: 'They referred to an IRB decision that forced to exclude those trial participants that initially gave consent but loose the decision making competence over the course of the trial due to disease progression'

Authors’ response: We agree that this sentence was difficult to understand and revised it accordingly. The revised version is:

“One example given by interviewees was an IRB decision that required the exclusion from the trial of those participants who had initially consented but lost capacity during the trial due to disease progression. Other IRBs allowed these participants to remain in the trial.”

Reviewer comment: Are the potential response strategies the same as the mitigation measures mentioned in the abstract? I would make this clearer and ensure that the language used to describe recommendations/response strategies/mitigation factors is consistent.

Authors’ response: Yes, we used to address the same topic. To avoid confusion we replaced mitigation strategies with response or potential response strategies.


Discussion
Reviewer comment: More direct discussion of the results would be beneficial here, including an explanation of how they link to the informed consent process. I would summarise the results first, reflect upon them, situate your findings within the wider context of why informed consent and trial prioritisation is important and then present your recommendations, whilst also drawing on other relevant literature.

Authors’ response: While incorporating the many helpful comments from both reviewer we also revised the Discussion section at several parts and we believe that it provides a comprehensive analysis of our results in relation to the informed consent process and trial prioritization, while also drawing upon relevant literature. In response to your comment on the Discussion section, we would like to clarify our approach: We start with a direct discussion of results by initiating our discussion with the topic of informed consent, highlighting the practical limitations that can impact the conditions for a valid consent. We aimed to provide a comprehensive understanding of how factors like "time pressure," "isolation conditions," and "uncertainty about expected benefits and harms" can challenge the validity of consent, especially in the context of COVID-19 research. We emphasize the implications of these challenges not only on the protection of trial participants but also on the effective and efficient conduct of clinical research. We also discuss potential solutions, such as the secondary use of patient data under broad consent models and alternative consent strategies, drawing parallels with the RECOVERY Study and other international examples. We then delve into the broader implications of our findings, discussing the societal impact of recruitment challenges and the potential for biased sampling. We also highlight the need for ethical, legal, and social discussions to address modifications of consent procedures in pandemic situations.

Reviewer comment: The example of research in LMICs feels a little out of place as the rest of the manuscript is focussing on Germany. The point about varying contexts could either be developed in more detail, or the reference removed.

Authors’ response: Thank you for highlighting the inclusion of the example related to research in LMICs. The primary intention behind referencing the study by Singh et al. (2021) was to illustrate the recommendations and perspectives of our interview participants. Our interviewees suggested that secondary use of patient data under broad consent models might address some of the challenges they identified. The reference to the study in the context of LMICs was not to shift the focus to LMICs per se, but rather to provide an exemplary discussion on the ethical considerations surrounding informed consent during public health emergencies, as highlighted by our interviewees. While the geographical context of the example is LMICs, the core message we aimed to convey is the potential solution to the challenges of informed consent, as recommended by our interview participants. We are not aware of another reference that describe such a scenario in, for example, Europe. We added text to comparise with Germany's national broad consent model to further emphasize the variability and adaptability of consent procedures, depending on specific challenges and recommendations.

Nice reflection on the limitations of the study – thank you.


Minor points
Reviewer comment: A thorough grammar check is required - there are a lot of small errors throughout the manuscript eg first sentence of the introduction should read ‘parts’ not ‘part’ and ‘too little patients’ should read ‘too few’

Authors’ response: We corrected these examples and conducted a thorough grammar check.

Please also check the use of tense throughout, as it is not consistent.

Authors’ response: We went through the manuscript to check the use of tense.

Reviewer comment: Can you explain what a ‘member of public funder is’? Does this mean a ‘member of a public funding body’ or ‘public donor’?

Authors’ response: Yes, it means a member of a public funding body. We changed this throughout the manuscript.

Reviewer comment: Whilst the meaning of quotations should not be changed or edited, it would be advisable to edit them to make them easier to read.

Authors’ response: We edited all quotations to make them easier to read.

Reviewer comment: The use of the word ‘incapable’ would be better amended to ‘unable’.

Authors’ response: We changed the wording accordingly.

Reviewer comment: The age ranges in the table need to be modified - 45 appears in both 35-45 and 45-50

Authors’ response: We thank the reviewer for pointing to this error. We revised the age ranges accordingly and now list 35-44.

Reviewer comment: Principal investigator rather than ‘principle’ investigator

Authors’ response: We changed the wording accordingly.

Reviewer comment: I am not familiar with the context thus cannot be sure, but would it be possible for the ‘non-PI investigator’ or the ‘member of public funder’ to be identified? There is only one in each category, so I wonder if readers familiar with the context and the networks of the authors would be able to identify them

Authors’ response: As we do not provide the full transcripts, but individual phrases and we do not relate the interviewees’ characteristics to the interviews, we expect that it would be rather difficult to identify single interviewees.
