# Supplementary material for: Informed consent and trial prioritization for clinical studies during the COVID-19 pandemic. Stakeholder experiences and viewpoints
Source: PLoS One. 2024 Apr 30;19(4):e0302755. doi: 10.1371/journal.pone.0302755 (PMC11060594; doi:10.1371/journal.pone.0302755)
Supplement: Attachment — Submitted filename: Responses to reviewer comments final.docx [file pone.0302755.s005.docx]

Authors’ responses to reviewer comments

Additional Editor Comments:

Reviewers comments are adequately addressed.

Consider moving the content under heading ‘demographics’ to the methods section.
-Add – ‘We conducted 21 semi-structured in-depth interviews with 21 participants to reach thematic saturation. Altogether, we contacted and invited 51 stakeholders. Our sample included physicians working in clinical research (principal investigators and non-principal investigators), heads of clinical departments that were responsible for the enrollment of patients with COVID-19 in clinical studies, representatives from clinical study centers, members of research ethics committees, and a member of a public funding body. For complete demographics see table 1.’ To data collection

Authors’ response: We moved the content accordingly.

-Add- ‘For the first topic informed consent we identified three main themes: consent challenges, impact of consent challenges on clinical research, and potential strategies for consent challenges. For the second topic prioritization of clinical studies, we identified two main themes: perceived benefit of prioritization, potential strategies for prioritization. All main themes were further specified with first and second level subthemes (see table 2 for an overview). In the following we give a narrative overview of the identified main and subthemes together with quotes for selected subthemes. The corresponding original quotes from the interviews for all subthemes can be found in supplementary table S2.’ To data analysis

Authors’ response: We moved the content accordingly but kept the last two sentences in the results section as they directly introduce the following results sections.

Minor:
‘…consenting procedureswith every COVID-19 patient’ under the Discussion section. Kindly revise to – ‘…consenting procedures with every COVID-19 patient’

Authors’ response: We changed the text accordingly.
